# An efficient simulated annealing algorithm for the RNA secondary structure prediction with Pseudoknots

**DOI:** 10.1186/s12864-019-6300-2

**Published:** 2019-12-27

**Authors:** Zhang Kai, Wang Yuting, Lv Yulin, Liu Jun, He Juanjuan

**Affiliations:** 1 School of Computer Science, Wuhan University of Science and Technology, Wuhan, 430081 China; 2 Hubei Province Key Laboratory of Intelligent Information Processing and Real-time Industrial System, Wuhan, 430081 China

**Keywords:** RNA secondary structure, Pseudoknot, Simulated annealing algorithm, Minimum free energy

## Abstract

**Background:**

RNA pseudoknot structures play an important role in biological processes. However, existing RNA secondary structure prediction algorithms cannot predict the pseudoknot structure efficiently. Although random matching can improve the number of base pairs, these non-consecutive base pairs cannot make contributions to reduce the free energy.

**Result:**

In order to improve the efficiency of searching procedure, our algorithm take consecutive base pairs as the basic components. Firstly, our algorithm calculates and archive all the consecutive base pairs in triplet data structure, if the number of consecutive base pairs is greater than given minimum stem length. Secondly, the annealing schedule is adapted to select the optimal solution that has minimum free energy. Finally, the proposed algorithm is evaluated with the real instances in *PseudoBase.*

**Conclusion:**

The experimental results have been demonstrated to provide a competitive and oftentimes better performance when compared against some chosen state-of-the-art RNA structure prediction algorithms.

## Background

RNA is a linear molecular compound formed by polymerization of ribonucleotides with phosphodiester bonds, the ribonucleotides are composed of phosphoric acid, ribose and bases. The RNA sequence consists of Adenine (A), Uracil (U), Guanine (G) and Cytosine (C), the four-base arrangement allows RNA to have a variety of functions that can play great role in genetic coding, translation, regulation, and gene expression. The search for the secondary structure of RNA sequence has been widely used as the first step to understand biological functions [1].

Pseudoknot is a special RNA secondary structure that is found in many important biologically molecules [2, 3], it usually contains not well-nested base pairs. These non-nested base pairs make the presence of pseudoknots in RNA sequences more difficult to be predicted by dynamic programming, which use a recursive scoring system to identify paired stems. The general problem of predicting minimum free energy (MFE) structures with pseudoknots is NP-complete problem [4]. In general, researchers apply the principle of MFE to evaluate RNA secondary structure. When the RNA sequence is freely folded in space to form the secondary structure of MFE under fixed experimental conditions, the change is stopped, meanwhile, the stable state of the RNA sequence is formed. For the calculation of the free energy of RNA secondary structure, the stem energy is defined as a negative, the energy of loop is defined as a positive, and the free single strand does not participate. Deng found that the molecular free energy is related to a single complementary base pair, but adjacent base pairs also affect the free energy calculation of the molecule [5]. In the secondary structure prediction, if the free energy calculation of each part does not affect each other, the free energy of the entire structure is accumulated form the energy of each part, and the calculation principle is shown in Eq. (1).

1 $$ \varDelta G=\sum \varDelta {G}_S+\sum \varDelta {G}_H+\sum \varDelta {G}_I+\sum \varDelta {G}_B+\sum \varDelta {G}_M+\sum \varDelta {G}_P+\varDelta \delta $$

In the above formula, *ΔG*_*S*_ means the stem free energy; *ΔG*_*H*_, *ΔG*_*I*_, *ΔG*_*B*_, and *ΔG*_*M*_ represent the free energy of hairpin, internal, bulged, and multi-branch loop, respectively; *ΔG*_*P*_ represent the pseudoknot free energy, which is generally split into loop for calculation to simplify the calculation process; *Δδ* is a threshold set to balance the error during the experiment process. After the RNA secondary structure is calculated in the Eq. (1), researcher can objectively evaluate whether the current structure is stable by numerical changes.

At present, existing algorithms for the prediction of RNA secondary structure with pseudoknots can be classified into two categories. The first category is dynamic programming (DP) based approaches. DP is the initial computational approach used to predict RNA structure [6]. The idea of dynamic programming is to divide a complex problem into many simple sub-problems to facilitate their treatment [7]. Combining the DP idea with the principle of MFE, researchers have proposed many RNA secondary structure prediction algorithms. Rivas and Eddy [8] proposed pknots-RE algorithm that can predict RNA sequence with pseudoknot structure. Dirks and Pierce [9] proposed NUPACK algorithm which calculate a series of recursion probabilities that can be used to compute base-pairing probabilities with or without pseudoknots. However, these algorithms are very time-consuming to predict long-chain sequence, and its maximum predictive sequence length cannot exceed 150.

The second category is Heuristic based approaches, which can handle long RNA sequences and obtain high quality feasible solution efficiently [10]. Ren et al. [11] proposed HotKnots to build up candidate secondary structures by adding substructures one by one to partially formed structures. Zuker et al. [12] and Turner et al. [13] integrate thermodynamic model into their algorithms to search for secondary structure with minimal free energy. SARNA-predict-pk [14] algorithm is an extended version of SARNA-Predict [10] which predicts RNA secondary structures with pseudoknots. This algorithm employs a new thermodynamic model that was described by Rastegari and Condon [15] and implemented in the HotKnots software. The model can be used to evaluate RNA sequences with pseudoknots. IPknot [16] algorithm proposed a computational method for predicting RNA secondary structures with pseudoknots based on maximizing the expected accuracy of a predicted structure. Iterative HFold [17] takes as input a pseudoknot-free structure, and produces a possibly pseudoknotted structure whose energy is at least as low as that of any (density-2) pseudoknotted structure containing the input structure. It leverages strengths of earlier methods, namely the fast running time of HFold, a method that is based on the hierarchical folding hypothesis and the energy parameters of HotKnots V2.0. Fatmi et al. [18] proposed a new algorithm that combines between the Greedy Randomized Adaptive Search Procedure (GRASP) and the Genetic Algorithm (GA) principle. This method repeats a process consisting of two phases: the construction phase and the local search phase. During the construction phase, a list of feasible solutions is iteratively constructed. The local search phase comes with the wake of the construction step; it aims to improve the solution obtained from the first phase by launching a local search to find the local optimum solution.

In this paper, a novel efficient simulated annealing (SA) algorithm is proposed to predict RNA secondary structure with pseudoknot. Firstly, an efficient base pairing method is designed, which is based on the minimum stem length and the minimum loop length, and a completed conflict resolution is provided for the conflicting bases; Then a simple and effective fitness function is proposed, and the number of stem and the total number of base pairs of the RNA sequence is used as metrics for evaluating the secondary structure of RNA; Finally, the annealing schedule is selected to systematically decrease the temperature as the algorithm proceeds, the final solution is the structure with MFE. In this paper, eighteen test sequences are randomly selected from the *PseudoBase* [19], and the results are compared with other leading prediction algorithms such as HotKnots [11], IPknot [16], TT2NE [20], CombFold [21], RnaStructure [22], CyloFold [23] and RNAflod [24] which shows, the effectiveness of our algorithm.

## Methods

**Fig. 1:**
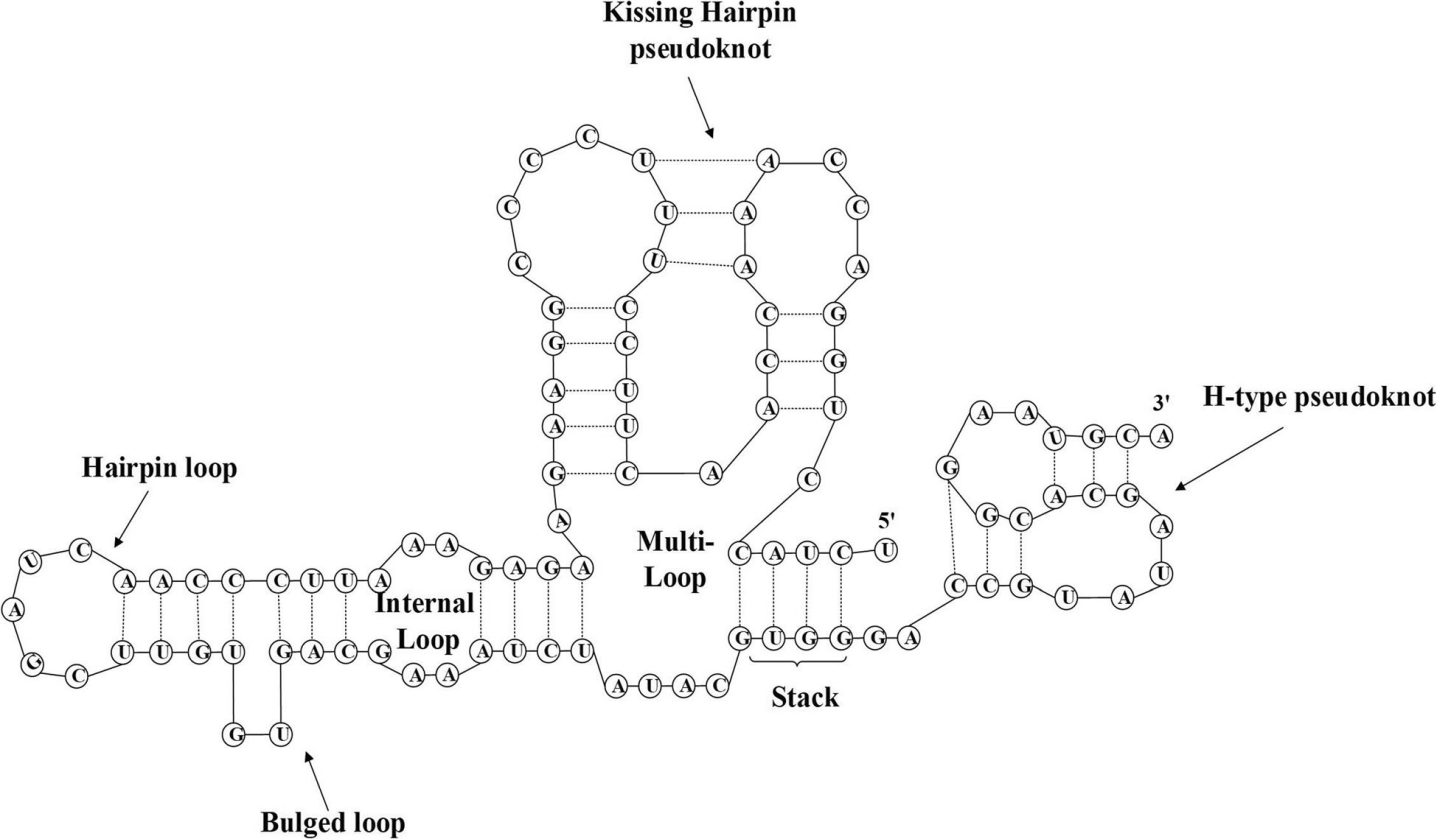
RNA Secondary Structure and Substructures

The RNA secondary structure folds itself by forming hydrogen bonds between G-C, A-U, and G-U. Therefore, the prediction of all hydrogen connections among the primary structure of the sequence become the first in predicting RNA secondary structure. Many components can be identified in the secondary structure, such as stem, hairpin loop, multi-branched loop or multi-loops, bulge loop, internal loop, and pseudoknot, as shown in Fig. 1.

### Definition

For a given RNA sequence *X* = 5′-*x*_1_*x*_2_…, *x*_*i*_, … *x*_*n*_-3′ of length *n*, *i* is defined as the initial index of the current base and *Y*(*X*) is the mapping string of consecutive complementary base pairs of *X*, *Y*(*X*) = (*y*_1_, *y*_2_, …, *y*_*i*_, …, *y*_*n*_), *y*_i_ is assigned to be *j*, if base *x*_*i*_ bond with base *x*_*j*_, as shown in Eq. 2.

2 $$ {y}_i=\Big\{{\displaystyle \begin{array}{c}j,\mathrm{if}\;{x}_i\;\mathrm{paired}\kern0.17em \mathrm{with}\;{x}_j\\ {}i,\mathrm{else}\kern6em \end{array}} $$

**Fig. 2:**
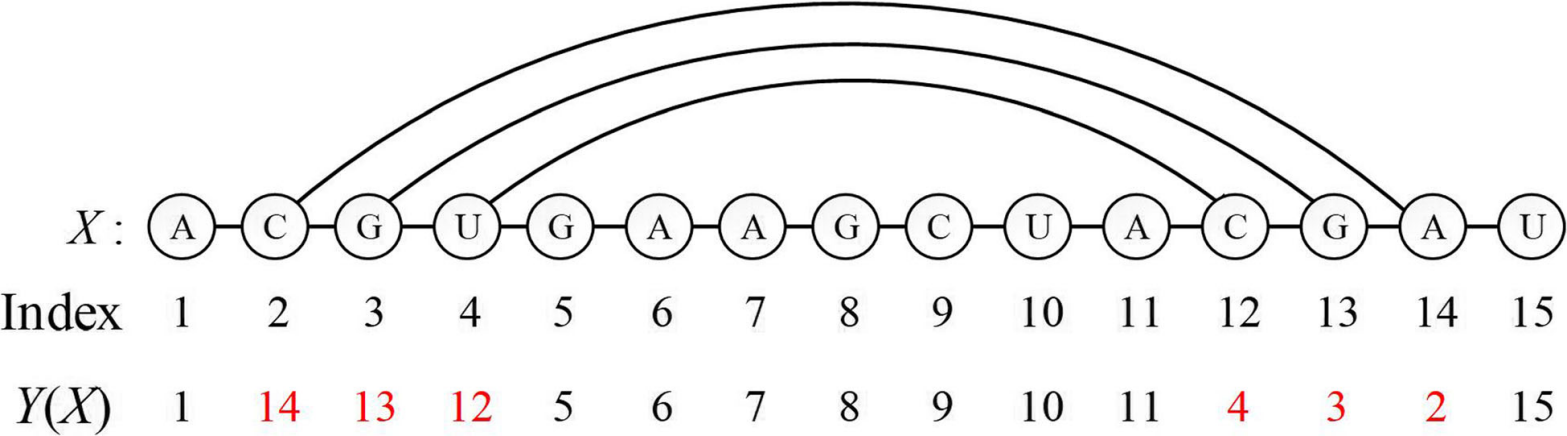
One of the mapping string *Y*(*X*) for sequence *X*

As shown in Fig. 2, when the base is paired, the sequence numbers of the paired bases are exchanged and stored in *Y*(*X*), then *Y*(*X*) = (1, 14, 13, 12, 5, 6, 7, 8, 9, 10, 11, 4, 3, 2, 15). Each mapping string *Y*(*X*) is a candidate solution, the solution with MFE is the optimal solution, which is the most stable secondary structure.

In order to better simulate the folding process of RNA secondary structure in the program, we define each part of the RNA secondary structure as follows:

**Definition 1**: *X* = 5′-*x*_1_*x*_2_…*x*_*n*_-3′, *x*_*i*_ ∈ {A, U, G, C}, Sequence *X* is called an RNA sequence of length *n*.

**Definition 2 (stem)**: *x*_*i*_*x*_*i* + 1_…*x*_*i* + *k*-1_ and *x*_*j*-*k* + 1_…*x*_*j*-1_*x*_*j*_ is two sub-segments in sequence *X*, (*x*_*i*_, *x*_*j*_) ∈ W = {(A, U), (U, A), (G, C), (C, G), (G, U), (U, G)}, 1 ≤ *i < j* ≤ *n*, *j* − *i*≥ 3, then the structure of consecutive base pairing by {(*x*_*i*_, *x*_*j*_), (*x*_*i* + 1_, *x*_*j*-1_),…, (*x*_*i* + *k*-1_, *x*_*j*-1_)} is called the stem of length *k* (*k* ≥ 2). To simplify calculations, stem can be expressed as a *m*_*i*_ = (*i*, *j*, *k*), where parameters *i* and *j* are the index of beginning base and ending base, and parameter *k* is the length of this stem.

**Definition 3 (hairpin Loop)**: There must be at least *MinLoop* (*MinLoop* ≥ 3) unpaired bases in any hairpin loop structure.

**Definition 4 (consecutive complementary base paired set)**: The complete RNA secondary structure of a sequence *X* is called a consecutive complementary base pair set, recorded as *M(X)*, *M*(*X*) = (*m*_1_, *m*_2_,…, *m*_*i*_, …,*m*_*n*_). Each m_*i*_ represents a stem, according to the above definition, any *m*_*i*_ can be recorded as (*i*, *j*, *k*). In the sequence *X*, the secondary structure formed by the pairing of *M(X)* is represented by *Y(X)*.

**Fig. 3:**
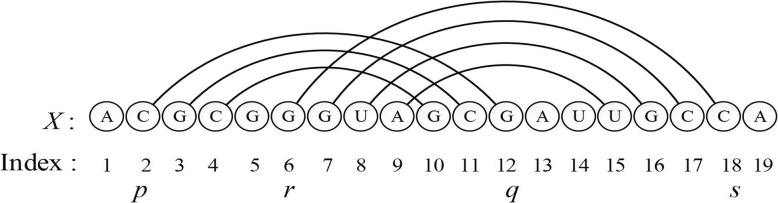
A arc representation for pseudoknot structure

**Definition 5 (pseudoknot)**: ∀ *x*_*p*_, *x*_*q*_, *x*_*r*_, *x*_*s*_, ∈ *X*, (*x*_*p*_, *x*_*q*_), (*x*_*r*_, *x*_*s*_) ∈ W, and the number of four bases in *X* satisfies 1 ≤ *p < r < q < s* ≤ *n* or 1 ≤ *r < p < s < q* ≤ *n*, then the structure formed by these two base pairs is called a pseudoknot structure, as shown in Fig. 3.

According to the above definition, the secondary structure prediction problem with pseudoknot can be converted to find the number of stems in all possible stem of the *X* sequence. These stems are so unique that secondary structure formed by their base complementarity has MFE state. Thus, an efficient Prediction algorithm of RNA secondary structure with pseudoknot based on SA (PRSA) is proposed. 

### Set of K consecutive base pairs

**Fig. 4:**
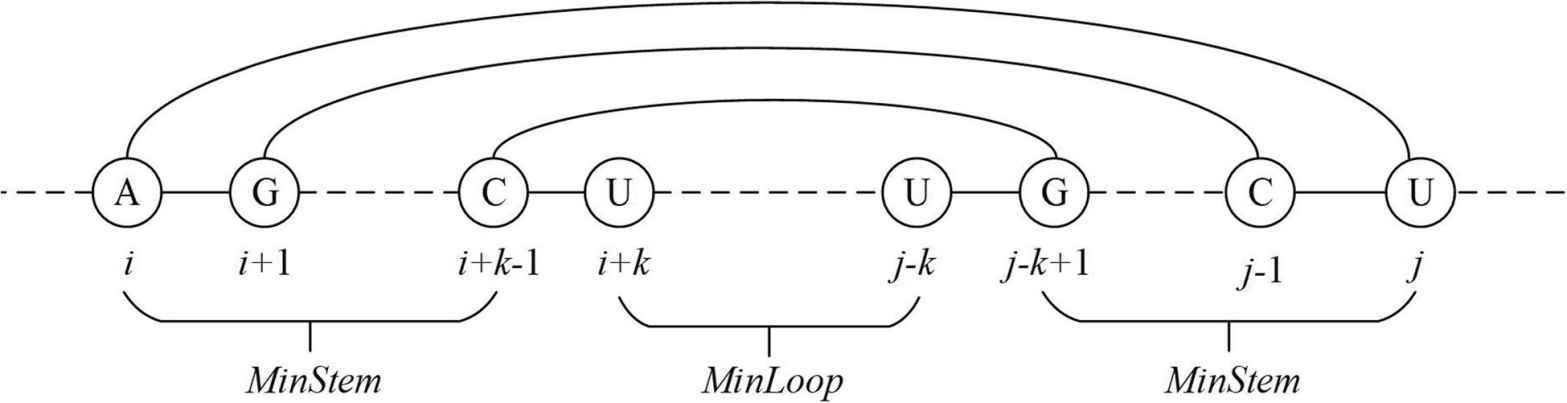
Consecutive paired *MinStem* and unpaired *MinLoop*

Since single base pairs cannot contribute to the reduction of free energy, the PRSA algorithm considers consecutive base pairs. In order to find all the stem structures, we defined the minimum stem length (*MinStem* ≥ 2) and the minimum loop length (*MinLoop* ≥ 3) parameters, as shown in Fig. 4.

After initially setting the parameters *MinStem* and *MinLoop*, all the reasonable *m*_*i*_ can be calculated. Parameters *i*, *j* and *k* need to satisfy the following three constraints:

3 $$ 1\le i\le n-2\ast MinStem- MinLoop+1 $$

4 $$ i+2\ast MinStem+ MinLoop-1\le j\le n $$

5 $$ MinStem\le k\le \frac{j-i- MinLoop+1}{2} $$

For example, Mengo_PKB is an RNA molecule from the *PseudoBase*, whose sequence is 5^′^ − ACGUGAAGGCUACGAUAGUGCCAG − 3^′^. Let *MinStem* and *MinLoop* be 3, all possible triplets (*i*, *j*, *k*) are (2,14,3), (2,14,4), (2,20,3), (3,13,3), (3,21,3), (8,22,3), (9,19,4), (10,18,3), (11,20,3). The pseudo code of calculation consecutive base pairs is shown as Algorithm 1.

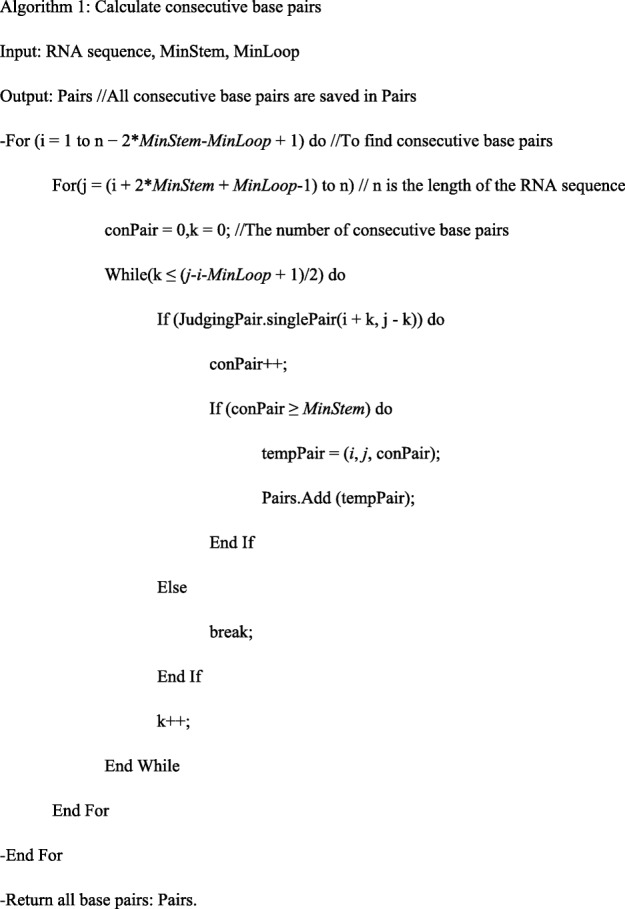



But in all base pairs, the same position of bases may have different consecutive base pair numbers, we need to merge these same positions. Like the above Mengo_PKB sequence, the set of base pairs after the merge is (2, 14, (3, 4)), (2, 20, (3)), (3, 13, (3)), (3, 21, (3)), (8, 22, (3)), (9, 19, (3, 4)), (10, 18, (3)), (11, 20, (3)). The pseudo code that saves the merged result to the K consecutive base pair set is shown in Algorithm 2.

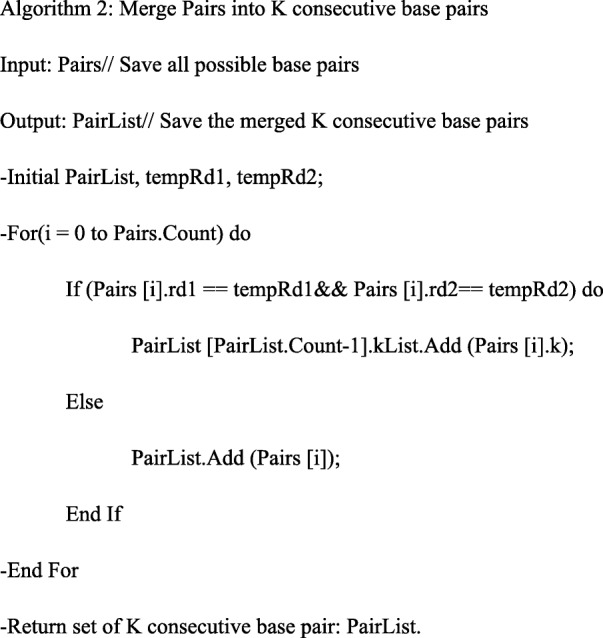



As known that most predicted algorithms require more effort to calculate the MFE structure after calculating the free energy of the current prediction, which makes their algorithm converge very slowly. A pool of candidate structures is generated by constructing a set of K consecutive base pairs, which makes the PRSA algorithm converge faster than other prediction algorithms. This also makes each iteration more valuable because each iteration generates a new structure from the candidate pool.

### Neighbor state and its conflict

When the secondary structure prediction is performed on any of the RNA molecules, the PRSA algorithm would first calculate the K consecutive base pair set by parameter preprocessing, and then generate a neighbor state through a random function in the simulated annealing algorithm.

**Fig. 5:**
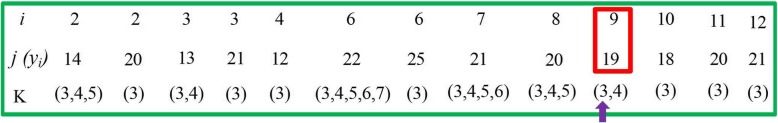
K consecutive base pairs set of TMEV molecules

Taking the TMEV molecule as an example, after the preprocessing process of the upper section ‘Set of K consecutive base pairs’, a K consecutive base pairs set of TMEV molecules is obtained, as shown in Fig. 5.

Divided according to the base start position and end position of stem, this set contains 13 elements. Since the base start and end positions of the stem are the same, different stem lengths may exist, so the algorithm determines one stem by generating two random numbers. The first random number is between 1 and 13, and the second random number is related to its corresponding set of K consecutive base pairs.

**Fig. 6:**
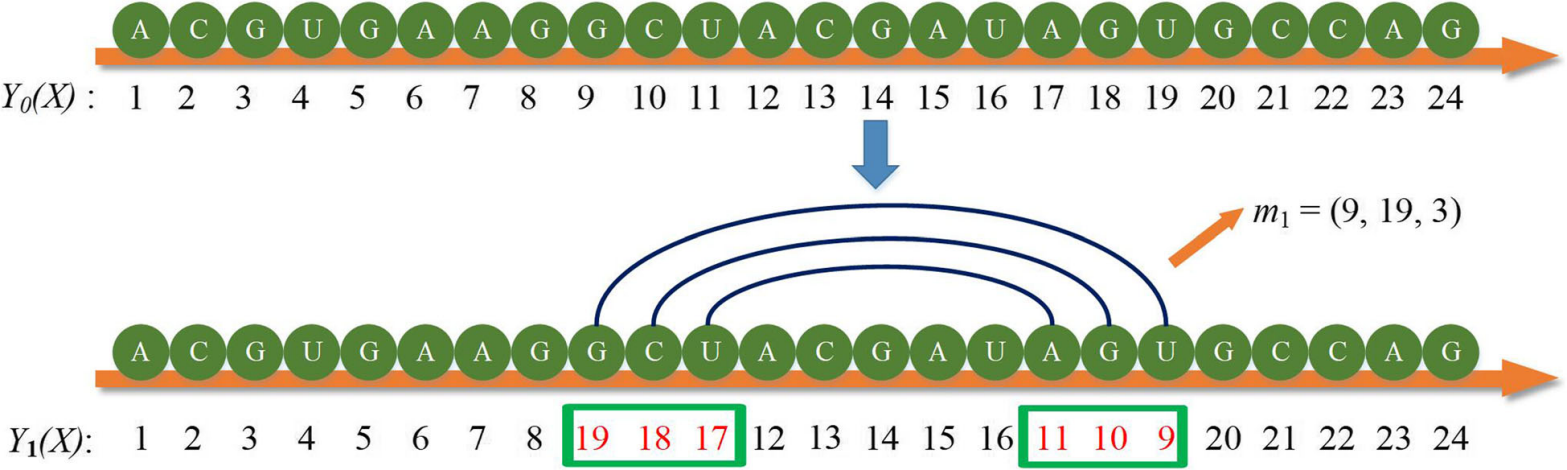
*m*_1_ base number exchange process

**Fig. 7:**
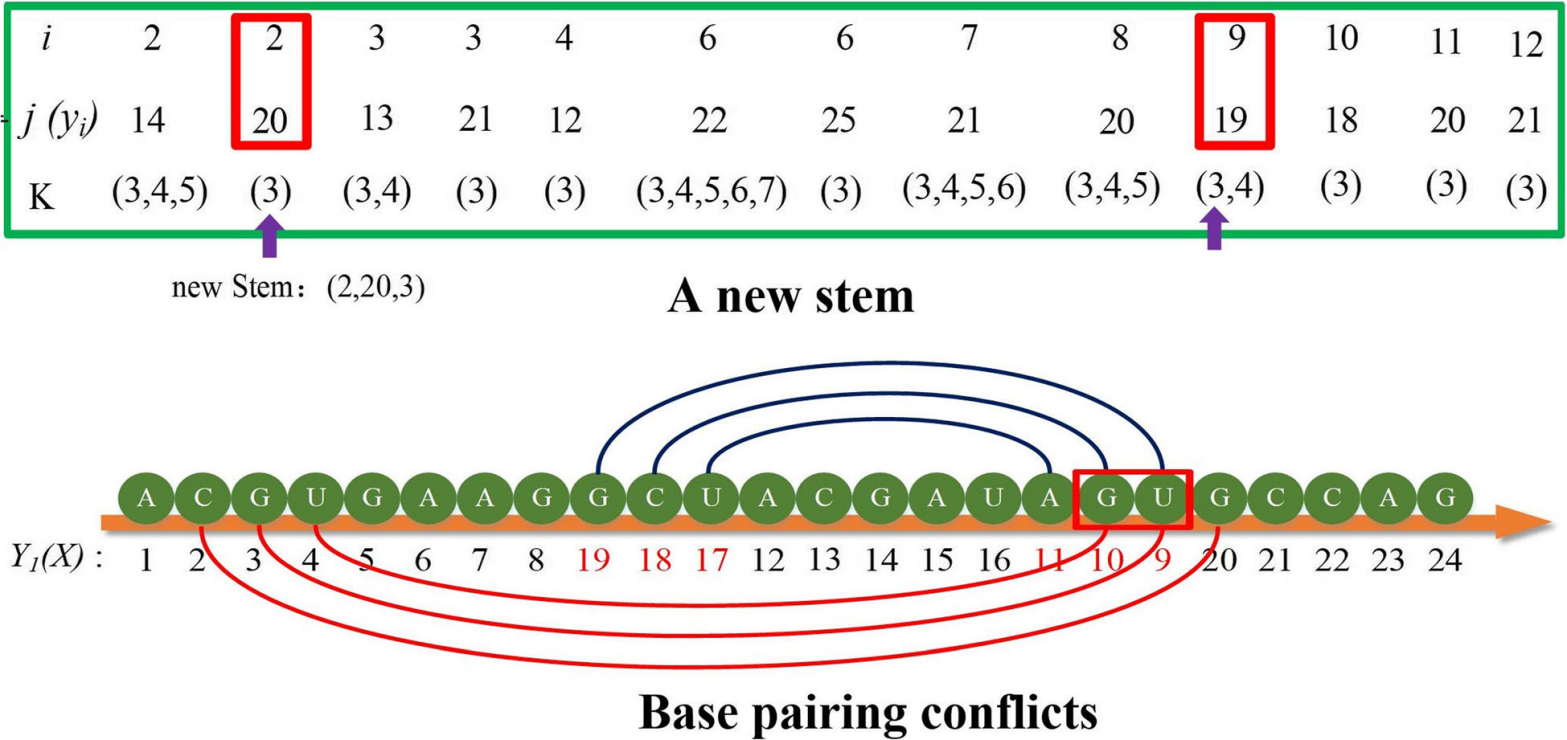
New neighboring state generation process

**Fig. 8:**
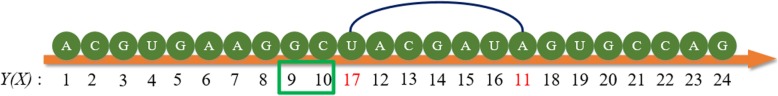
Remove base pairing conflicts

**Fig. 9:**
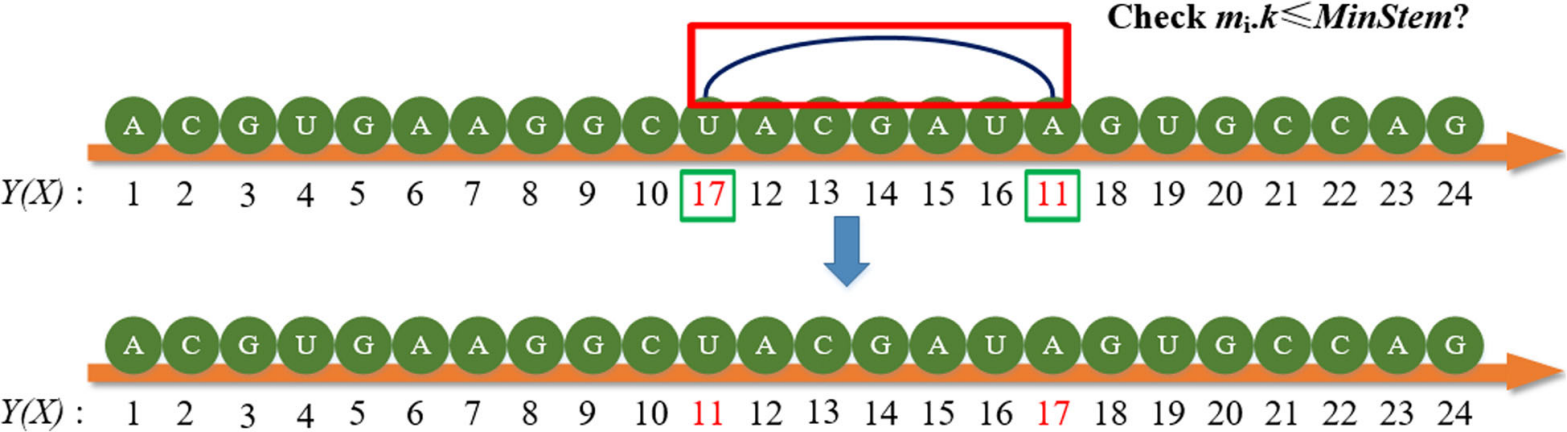
Check the rationality of remaining *m*_i_

For example, take two random values as 10 and 1, respectively. At this time, *m*_1_ = (9, 19, 3), a local RNA secondary structure is formed. In order to be recorded in the programming, this section of the algorithm has been processed in 4 steps:

(1) The paired base numbers are exchanged as shown in Fig. 6, *m*_1_ is added to the consecutive base pair set *M(X)*, at this time *M(X)* = {*m*_1_ = (9, 19, 3)}, and the secondary structure corresponding to *M(X)* is represented by *Y*_1_*(X)*.

(2) A randomly generated *m*_*i*_ that may conflict with elements in the set *M(X)*. When the algorithm program performs the next iteration of the loop, a new stem *m*_2_ = (2, 20, 3) is generated. At this time, a base pairing conflict occurs, that is, the bases originally numbered 18 and 19 have been paired with the bases at other positions, and the base complementary pairing conflicts are shown in Fig. 7.

(3) If there is a conflict, the position number of the conflicting base is exchanged again to remove the conflict, and the *m*_1_ in the *M(X)* is updated, and the schematic diagram of removing the base pairing conflict is shown in Fig. 8. The *M(X)* is updated to {*m*_1_ = (11, 17, 1)} after removal.

(4) Determine whether the updated *m*_*i*_ meets the constraint. If it does not, remove it; if it does, it will not be considered. When the constraint is initialized, the algorithm program sets the minimum length of the stem to be no smaller than *MinStem*. Assume that the initial value of *MinStem* is 3, therefore, the remaining pairing mode of *m*_1_ needs to be removed, and the element is deleted from *M(*X), and *M(X)* is an empty set. The operation process is shown in Fig. 9.

**Fig. 10:**
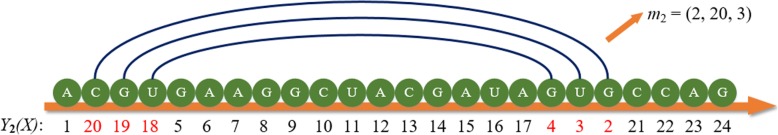
*m*_2_ base number exchange

After the conflicts and constraints are resolved, the base pairing is performed in the new stem and added to *M(X)*, as shown in Fig. 10. At this time, *M(X)* = {*m*_2_ = (2, 20, 3)}, the secondary structure corresponding to *M(X)* is represented by *Y*_2_*(X)*, and *Y*_2_*(X)* is the neighbor state of *Y*_1_*(X)*.

### Fitness function

For most MFE based RNA secondary structure prediction algorithm, the complex thermodynamic model is often used to evaluate candidate solutions [21]. However, there is no useful information to guide the candidate solution to find lower neighbor energy state. Consequently, the convergence of these MFE based prediction algorithms is very slow. Actually, only the consecutive base pairs stem *∆G*_*S*_ provide negative free energy which contributes to the reduction of free energy. The stability of RNA sequence can also be approximately evaluated by consecutive base pairs stem.

Where *Group* is the number of stems of the secondary structure of the RNA sequence, *TP* is the sum of the number of all base pairs in the sequence, *TP* divided by *Group* is the average number of base pairs (*AP*), *PG* is the predicted number of pseudoknots by the algorithm, *MG* is the expected number of pseudoknots, and *k* is the length of the stem. The evaluation function for random candidate *M*(*X*) can be seen in the following Equation:

6 $$ F\left(M(X)\right)=\Big\{{\displaystyle \begin{array}{cc} TP\times A{P}^2,& PG\le MG\\ {} TP\times A{P}^2\times \frac{Group- PG}{Group},& PG> MG\end{array}} $$

7 $$ TP=\sum \limits_{i=1}^n{m}_i.k $$

8 $$ AP=\frac{TP}{Group} $$

**Fig. 11:**
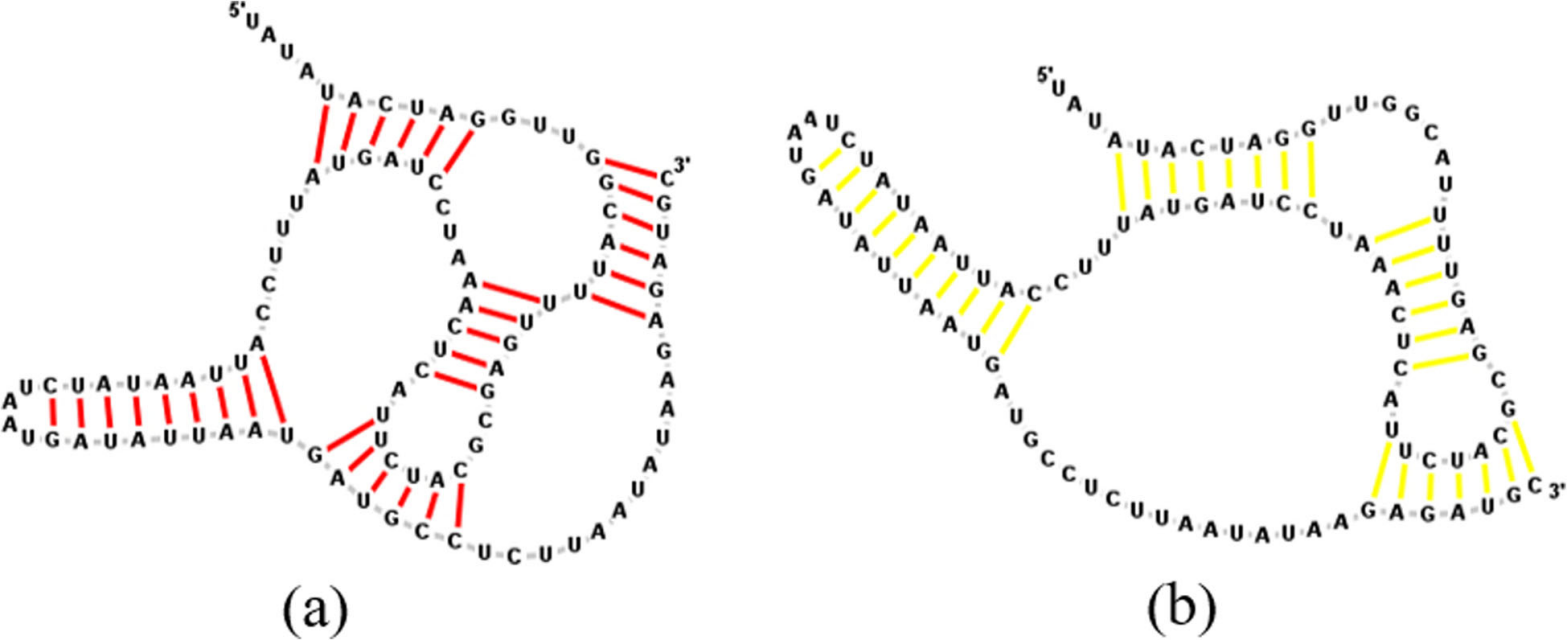
Two different secondary structures of BCRV1

The two structures of the BCRV1 molecule are evaluated using the custom fitness function,

*M*_1_*(X)* = {*m*_1_ = (5,47,6), *m*_2_ = (14,80,6), *m*_3_ = (20,38,5), *m*_4_ = (26,98,7), *m*_5_ = (53,74,9)}, as shown in Fig. 11a; *M*_2_*(X)* = {*m*_1_ = (4,48,8), *m*_2_ = (19,39,6), *m*_3_ = (26,98,7), *m*_4_ = (52,75,10)}, as shown in Fig. 11b. We produce the images of RNA structure with jViz. Rna [25].

After evaluation, the calculated data of the secondary structure of BCRV1 molecule are shown in Table 1. According to the fitness function values of the two structures, it indicates that *M*_2_ is better than *M*_1_.

**Table 1:** Evaluation results

Structure	MG	PG	Group	TP	AP	F(*M*(*X*))
*M*_1_(*X*)	1	2	5	33	6.6	862.49
*M*_2_(*X*)	1	1	4	31	7.75	1861.94

### Overall algorithm

The PRSA algorithm initializes the parameters to determine the constraints of the RNA sequence, thereby calculating a set of K consecutive base pairs. According to this set, the neighbor state is randomly generated, and the custom fitness function is adopted to evaluate the quality of the current solution (*CurrentPairs*) and the previous generation solution (*MaxPairs*). If the *CurrentPairs* performs better, it would replace the *MaxPairs* directly. Otherwise, it will determine whether to accept the new pairing structure based on probability from Boltzmann distribution. The final predicted solution structure is stored in *MaxPairs*, which has MFE and includes pseudoknot. The pseudo-code of the overall algorithm is shown in Algorithm 3.

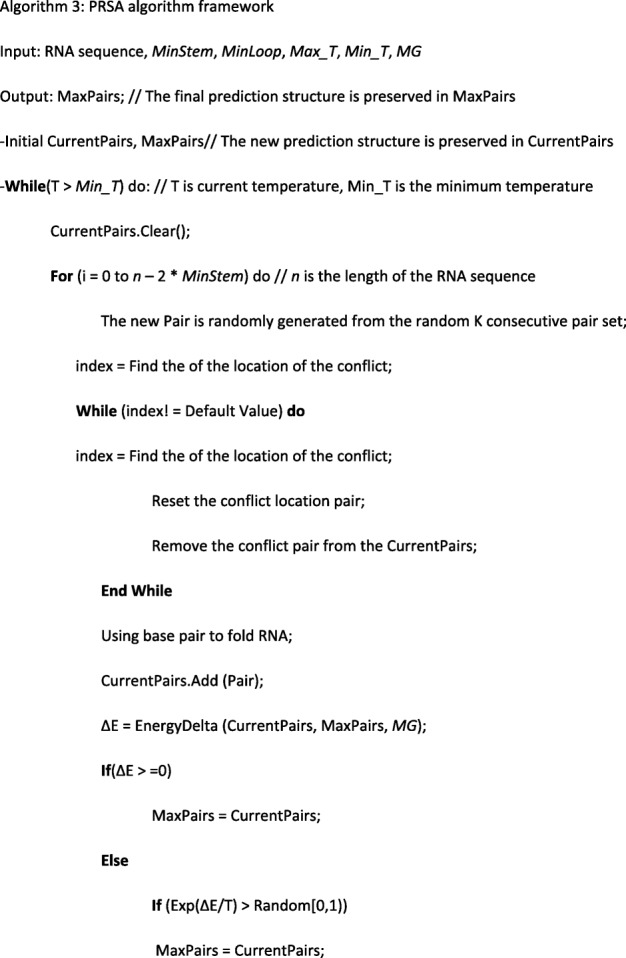



## Result

In section ‘method’, Predicting RNA secondary structures with pseudoknots is implemented using the PRSA algorithm. In the following, we first present the datasets, the exiting methods and accuracy measures we use for the evaluation of the algorithm, then the prediction performance of the PRSA algorithm is demonstrated by comparative experiments.

### Data sets

The eighteen benchmark instances from *PseudoBase* were used to test the proposed method. The characteristic of each sequence is shown in Table 2. The second column is the Abbreviation of the RNA sequence, the third column is the RNA PKB number, the fourth column is the RNA type, the fifth column is the sequence length and the last column is the number of base pairs in the known structure. The predicted structure should be similar to the base pairs of the known structure.

**Table 2:** Benchmark Instances from RNA *PseudoBase*

ID	RNA Abbreviation	PKB Number	RNA Type	Length (nt.)	Known bps
1	Mengo_PKB	PKB295	Viral 5 UTR	24	7
2	T4_gene32	PKB74	mRNA	28	11
3	HAV_PK1	PKB297	Viral 5 UTR	33	12
4	TEV_PK1	PKB277	Viral 5 UTR	35	11
5	IPCV1	PKB35	Viral tRNA-like	40	8
6	ScYLV	PKB281	Viral Frameshift	42	8
7	Ec_PK3	PKB51	tmRNA	46	14
8	Ec_PK4	PKB52	tmRNA	52	19
9	BEV	PKB128	Viral Frameshift	59	16
10	BaEV	PKB98	Viral Readthrough	62	15
11	VMV	PKB280	Viral Frameshift	68	14
12	ALFV	PKB350	Viral Frameshift	77	17
13	MVEV	PKB349	Viral Frameshift	80	18
14	SARS-CoV	PKB254	Viral Frameshift	82	26
15	FCiLV3	PKB395	Viral tRNA-like	109	37
16	BBMV3	PKB135	Viral tRNA-like	116	39
17	CVV3	PKB389	Viral tRNA-like	129	37
18	CCMV3	PKB136	Viral tRNA-like	134	45

### Accuracy measures

The prediction accuracy is calculated by comparing the predicted structure with the known structure. In order to assess the quality of the results produced, three evaluation criteria were used: sensitivity (SN%), specificity (SP%) and F-measure(%) [26]. The evaluation criteria are as follows:

9 $$ SN= TP\div \left( TP+ FN\right) $$

10 $$ SP= TP\div \left( TP+ FP\right) $$

11 $$ F- measure=2\ast SP\ast SN\div \left( SN+ SP\right) $$

Where TP represents the number of correctly predicted base pairs; FP represents the number of incorrectly predicted base pairs; FN represents the number of unpredicted base pairs compared with the known structure. When the prediction results are accurate, both SN and SP should be close to 100*%*.

### Comparison with existing methods

To better reflect the accuracy of the algorithm proposed in this paper, the computational results of the PRSA algorithm are compared with seven state-of-the-art algorithms, including HotKnots [11], IPknot [16], TT2NE [20], CombFold [21], RnaStructure [22], CyloFold [23] and RNAflod [24]. Among these algorithms, the HotKnots algorithm and the IPknot algorithm use heuristic ideas to predict the secondary structure. The names of the seven algorithms and the website links to the algorithm-based Web sites are listed in Table 3.

**Table 3:** State-of-the-art RNA structure predication algorithms

ID	Method	Website link
1	RnaStructure	http://rna.urmc.rochester.edu/RNAstructureWeb/
2	CyloFold	https://cylofold.ncifcrf.gov/
3	IPknot	http://rtips.dna.bio.keio.ac.jp/ipknot/
4	RNAflod	http://rna.tbi.univie.ac.at/cgi-bin/RNAWebSuite/RNAfold.cgi
5	CombFold	http://www.rnasoft.ca/cgi-bin/RNAsoft/CombFold/combfold.pl
6	HotKnots	http://www.rnasoft.ca/cgi-bin/RNAsoft/HotKnots/hotknots.pl
7	TT2NE	http://eole2.lsce.ipsl.fr/ipht/tt2ne/tt2ne.php

### Overall results

The comparisons of the proposed method with the other methods are shown in Tables 4, 5 and 6. If the value in the table is “#”, it means that the algorithm does not support the prediction of the length of the sequence, such as TT2NE. The results of the proposed method and the compared methods are all run 10 times for each sequence.

**Table 4:** Sensitivity Comparison Results

ID	#BP	Sensitivity (%)							
		1	2	3	4	5	6	7	PRSA
1	7	28.6	100.0	42.9	42.9	42.9	42.9	#	**100.0**
2	11	63.6	**100.0**	63.6	63.6	63.6	**100.0**	81.8	**100.0**
3	12	58.3	**100.0**	58.3	58.3	58.3	**100.0**	91.7	**100.0**
4	11	45.5	45.5	18.2	45.5	45.5	45.5	#	**90.9**
5	8	62.5	62.5	62.5	62.5	62.5	**100.0**	62.5	87.5
6	8	62.5	**100.0**	87.5	62.5	62.5	**100.0**	#	**100.0**
7	14	50.0	85.7	71.4	64.3	64.3	64.3	**100.0**	92.9
8	19	57.9	42.1	68.4	68.4	68.4	68.4	**100.0**	63.2
9	16	68.8	93.8	81.3	68.8	68.8	68.8	87.5	**100.0**
10	15	0.0	86.7	0.0	0.0	0.0	40.0	100.0	93.3
11	14	50.0	**100.0**	50.0	50.0	50.0	**100.0**	92.9	**100.0**
12	17	64.7	**100.0**	64.7	64.7	64.7	**100.0**	100.0	**100.0**
13	18	61.1	**100.0**	61.1	61.1	61.1	**100.0**	100.0	**100.0**
14	26	65.4	69.2	69.2	69.2	69.2	73.1	51.7	**84.6**
15	37	81.1	97.3	67.6	81.1	67.6	#	91.9	**100.0**
16	39	79.5	84.6	69.2	82.1	64.1	#	71.8	82.1
17	37	89.2	81.1	89.2	89.2	89.2	#	73.0	73.0
18	45	80.0	66.7	**84.4**	**84.4**	68.9	#	71.1	73.3
Average		59.4	84.1	61.6	62.1	59.5	78.8	86.7	**91.1**

The best Sensitivity values for each algorithm are shown in boldface

**Table 5:** Specificity Comparison Results

ID	#BP	Specificity (%)							
		1	2	3	4	5	6	7	PRSA
1	7	50.0	**100.0**	60.0	60.0	60.0	60.0	#	**100.0**
2	11	87.5	**100.0**	**100.0**	**100.0**	87.5	**100.0**	**100.0**	**100.0**
3	12	**100.0**	85.7	**100.0**	**100.0**	**100.0**	85.7	91.7	85.7
4	11	62.5	**100.0**	28.6	62.5	62.5	62.5	#	**100.0**
5	8	55.6	55.6	55.6	55.6	55.6	80.0	55.6	**100.0**
6	8	71.4	**88.9**	77.8	62.5	71.4	72.7	#	**88.9**
7	14	87.5	**100.0**	76.9	90.0	90.0	90.0	**100.0**	92.9
8	19	**100.0**	66.7	**100.0**	**100.0**	**100.0**	**100.0**	**100.0**	**100.0**
9	16	68.8	**100.0**	81.3	64.7	64.7	64.7	66.7	76.2
10	15	0.0	**81.3**	0.0	0.0	0.0	31.6	65.2	70.0
11	14	43.8	**73.7**	38.9	41.2	41.2	70.0	65.0	70.0
12	17	47.8	**73.9**	45.8	45.8	44.0	70.8	70.8	70.8
13	18	50.0	72.0	44.0	47.8	47.8	72.0	**75.0**	72.0
14	26	89.5	72.0	78.3	85.7	78.3	73.1	46.9	**100.0**
15	37	85.7	94.7	73.5	90.9	54.5	#	82.9	**97.4**
16	39	81.6	86.8	75.0	82.1	73.5	#	73.7	82.1
17	37	82.5	88.2	100.0	86.8	89.2	#	61.4	81.8
18	45	83.7	66.7	**88.4**	86.4	75.6	#	71.1	76.7
Average		69.3	83.7	68.0	70.1	66.4	73.8	73.2	**86.9**

The best Specificity values for each algorithm are shown in boldface

**Table 6:** F-measure Comparison Results

ID	#BP	F-measure (%)							
		1	2	3	4	5	6	7	PRSA
1	7	36.4	**100.0**	50.0	50.0	50.0	50.0	#	**100.0**
2	11	73.7	**100.0**	77.8	77.8	73.7	**100.0**	90.0	**100.0**
3	12	73.7	**92.3**	73.7	73.7	73.7	**92.3**	91.7	**92.3**
4	11	52.6	62.5	22.2	52.6	52.6	52.6	#	**95.2**
5	8	58.8	58.8	58.8	58.8	58.8	88.9	58.8	**93.3**
6	8	66.7	**94.1**	82.4	62.5	66.7	84.2	#	**94.1**
7	14	63.6	92.3	74.1	75.0	75.0	75.0	**100.0**	92.9
8	19	73.3	51.6	81.3	81.3	81.3	81.3	**100.0**	77.4
9	16	68.8	**96.8**	81.3	66.7	66.7	66.7	75.7	86.5
10	15	#	**83.9**	#	#	#	35.3	78.9	80.0
11	14	46.7	**84.8**	43.8	45.2	45.2	82.4	76.5	82.4
12	17	55.0	**85.0**	53.7	53.7	52.4	82.9	82.9	82.9
13	18	55.0	83.7	51.2	53.7	53.7	83.7	**85.7**	83.7
14	26	75.6	70.6	73.5	76.6	73.5	73.1	51.7	**91.7**
15	37	83.3	**96.0**	70.4	85.7	70.4	#	87.2	**98.7**
16	39	80.5	**85.7**	72.0	82.1	68.5	#	72.7	82.1
17	37	85.7	84.5	**94.3**	88.0	89.2	#	66.7	77.1
18	45	81.8	66.7	**86.4**	85.4	72.1	#	71.1	75.0
Average		66.5	82.7	67.1	68.8	66.0	74.9	79.1	**88.0**

The best F-measure values for each algorithm are shown in boldface

From Table 4, in terms of sensitivity, the proposed method provides the best results in nineteen sequences, of which 9 sequences predict 100%. In addition, there are 3 sequences predicting with sensitivities greater than 90%. In terms of specificity, the specificity of 8 sequences in Table 5 is more than 90%, including that the specificity of 6 sequences is 100%. For F-measure, there are 14 sequences exceeding 82%, including 9 sequences above 90%.

The proposed method has average sensitivity, specificity, and F-measure of 91.1, 86.9, and 88.0%, respectively. In addition, the average sensitivity of the proposed method is better than the CyloFold method by 7%, better than the TT2NE method by 4.4% and better than the HotKnots method by 12.3%. In case of the average of specificity, the proposed method is better than the CyloFold method by 3.2%, better than the TT2NE method by 13.7% and better than the HotKnots method by 13.1%*.* In case of the average of F-measure, the proposed method is better than the CyloFold method by 5.3%, better than the TT2NE method by 8.9% and better than the HotKnots method by 13.1%*.*

## Discussion and conclusion

**Fig. 12:**
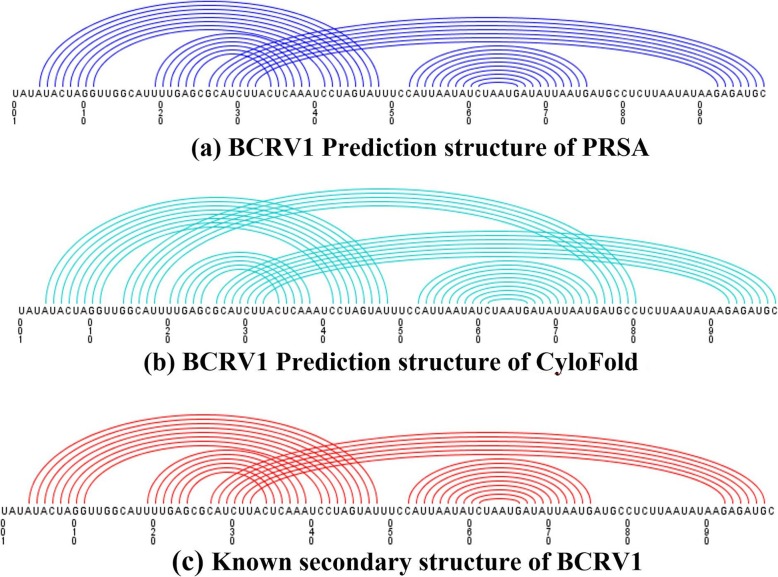
Comparison of predicted secondary structure by PRSA and CyloFold algorithm

According to Section ‘Accuracy comparison tests’, we can find that the PRSA algorithm has obvious advantages in the quality of the solution compared with other algorithms. Taking the BCRV1 molecule as an example, the sequence of this method is predicted by the PRSA algorithm and the CyloFold algorithm, respectively. The arc representation of the obtained secondary structure is shown in Fig. 12. It can be seen from the figure that the secondary structure predicted by the algorithm in this paper has become infinitely close to the real structure.

In this paper, we propose an efficient simulated annealing algorithm for the RNA secondary structure predicting with pseudoknots, combined with the evaluation function to compensate for the high time complexity of the free energy calculation model. The algorithm sets the *MinStem* and *MinLoop* parameters to determine the pseudoknot structure formed by the base pair cross-combination, and optimizes the pool of candidate solutions, thereby reducing the time cost of the algorithm. The custom evaluation function is used to improve the efficiency of RNA secondary structure prediction algorithms. Moreover, the performance of the PRSA algorithm is compared with state of art algorithms including eighteen *PseudoBase* benchmark instances, and the comparison results show that the PRSA algorithm is more accurate and competitive with higher sensitivity and specificity values.

However, as the size of RNA molecules becomes larger, this superiority will gradually disappear. The reason for the analysis may be that the algorithm for evaluating individuals is based on the average base pairs length rather than the standard thermodynamic model. As the length of the RNA molecule increases, the number of groups of complementary bases *M*(*X*) will become larger, so that the effect of average base-pairs on prediction results becomes weaker, the accuracy of the PRSA algorithm will be reduced. Besides, the parameter settings of the PRSA algorithm will also affect the prediction results, which will be studied further in the future.

## Data Availability

Pseudoknots sequencing data are available from the *PseudoBase* database (http://www.ekevanbatenburg.nl/PKBASE/PKB.HTML).

## Abbreviations

A: Adenine
C: Cytosine
DP: Dynamic Programming
G: Guanine
GA: Genetic Algorithm
GRASP: Greedy Randomized Adaptive Search Procedure
MFE: minimum free energy
NP: Non-deterministic Polynomial
RNA: Ribonucleic Acid
SA: Simulated Annealing
U: Uracil
